# Estimating the COVID-19 Prevalence in Spain With Indirect Reporting via Open Surveys

**DOI:** 10.3389/fpubh.2021.658544

**Published:** 2021-04-09

**Authors:** Augusto Garcia-Agundez, Oluwasegun Ojo, Harold A. Hernández-Roig, Carlos Baquero, Davide Frey, Chryssis Georgiou, Mathieu Goessens, Rosa E. Lillo, Raquel Menezes, Nicolas Nicolaou, Antonio Ortega, Efstathios Stavrakis, Antonio Fernandez Anta

**Affiliations:** ^1^Multimedia Communications Lab, etit, Technische Universität Darmstadt, Darmstadt, Germany; ^2^IMDEA Networks Institute, Madrid, Spain; ^3^Department of Statistics, University Carlos III de Madrid, Madrid, Spain; ^4^Departamento de Informática, University of Minho, Braga, Portugal; ^5^University of Rennes, Institut National de Recherche en Informatique et en Automatique, Centre National de la Recherche Scientifique, Institut de Recherche en Informatique et Systèmes Aléatoires, Rennes, France; ^6^Department of Computer Science, University of Cyprus, Nicosia, Cyprus; ^7^Independent Researcher, Nantes, France; ^8^University Carlos III de Madrid - Santander Big Data Institute, Madrid, Spain; ^9^Departamento de Matemática, University of Minho, Braga, Portugal; ^10^Algolysis Ltd, Limassol, Cyprus; ^11^Department of Electrical and Computer Engineering, USC Viterbi School of Engineering, Los Angeles, CA, United States

**Keywords:** COVID-19, pandemic, serology, survey, indirect reporting, sensing

## Abstract

During the initial phases of the COVID-19 pandemic, accurate tracking has proven unfeasible. Initial estimation methods pointed toward case numbers that were much higher than officially reported. In the CoronaSurveys project, we have been addressing this issue using open online surveys with indirect reporting. We compare our estimates with the results of a serology study for Spain, obtaining high correlations (R squared 0.89). In our view, these results strongly support the idea of using open surveys with indirect reporting as a method to broadly sense the progress of a pandemic.

## 1. Introduction

During the initial phases of the COVID-19 pandemic, progress tracking via massive serology testing has proven to be unfeasible. However, initial estimation methods suggested that the real numbers of COVID-19 cases were significantly higher than those officially reported (1). For instance, by April 30th, 2020, the number of confirmed fatalities due to COVID-19 in the US was 66, 028, and the number of confirmed cases was 1, 080, 303. However, with that number of fatalities the number of cases must have been no < 4, 784, 637, by simply using the Case-fatality Ratio (CFR) of 1.38% measured in Wuhan (2).

In the case of Spain, the discrepancy seems to be even higher. Preliminary studies point toward only one in 53 cases being reported during the first days of the pandemic (3). Although recent availability of massive testing has reduced this discrepancy, demographic statistics still indicate a degree of underreporting to this day, which can be seen among others in mortality numbers: all-cause mortality statistics in Spain point to two periods of significant excess of deaths in the country over the predicted values in 2020: March and April (44, 599 deaths in excess) and August to December (26, 186 deaths in excess) (4). These numbers contrast with the officially reported number of deaths due to COVID-19, which rests at 50, 837 (5). This discrepancy is corroborated in publications from official government authorities, which indicate an ongoing estimated underreporting of 20–40% (6).

A potential method to address this limitation is to use online surveys during the initial stages of pandemics. Online surveys can be deployed quickly and are cost-effective, but show potential weaknesses in sampling, confidentiality, and other ethical issues (7). In spite of these weaknesses, online surveys have already been successfully implemented in scenarios, such as influenza tracking (8).

In the CoronaSurveys project, Ojo et al. (9) we aim to track the progress of the COVID-19 pandemic using online, open, anonymous surveys with indirect reporting. Other recent articles have also suggested the use of surveys to monitor this pandemic, both for Spain (10, 11) and globally (12). However, to our knowledge, all surveys conducted in Spain have employed direct reporting only, asking participants about themselves. CoronaSurveys implements the network scale-up method of indirect reporting instead, allowing us to collect data on a wide fraction of the population with a small number of responses and in a very short time-frame (13). In this article, we compare the accuracy of CoronaSurveys with a gold standard: serology testing data collected by the Spanish government in the ENE-COVID study (14).

## 2. Methods

The survey deployed in the CoronaSurveys project can be answered via browser or mobile app. After the participant indicates the region (Spanish autonomous community) for which information will be provided, two additional questions are presented:

1. *How many people do you know in your area for which you know their health condition?* The answer to this question by participant *i* is the *Reach r*_*i*_.
2. *How many of those were diagnosed with or have symptoms of COVID-19?* The answer to this question by participant *i* is the *Cumulative Number of Cases c*_*i*_.

In the CoronaSurveys project we have focused on simplicity and brevity to maximize interest and retain users that would consistently provide data every few days. For that reason the total number of questions in the survey has been kept small at all times. Our approach yielded good initial results with about 200 responses per week. The survey has been promoted via social networks, direct contacts, and, more recently, with paid advertising.

To ensure total anonymity, the surveys are hosted on a private instance of LimeSurvey (15). Data is aggregated daily, and in this process the responses are shuffled so no single entry can be back-traced to its user. All the data is published in a public Github repository. The study design was reviewed and approved by the ethics committee of the IMDEA Networks Institute. The survey includes an informed consent.

Once the data is collected, we remove outlier responses. A response is considered an outlier if (1) *r*_*i*_ is outside 1.5 times the interquartile range above the upper quartile (which for the data in this paper means *r*_*i*_ > 175) or if (2) *c*_*i*_/*r*_*i*_ is > 1/3 (to exclude participants with an exceptionally high contact with cases). Although participants may choose to provide information for the whole country, in this paper we only consider responses in which participants provide information for their specific region. Hence, the data is aggregated by region for all participants, to obtain the estimator of COVID-19 prevalence $\left(\underset{i}{\sum }c_{i}\right)/\left(\underset{i}{\sum }r_{i}\right)$ (13).

## 3. Results

To assess the accuracy of this method in estimating the cumulative number of cases of COVID-19, we compare our cross-sectional survey estimates with the results of the serology study of Pollán et al. (14) for Spain. We exclude Ceuta and Melilla due to lack of data on our part. Conducted between April 27 and May 11, 2020, the serology study provides data for *n* = 61, 075 participants (0.1787 ± 0.0984% of the regional population, and 0.1299% of the national population). We consider as positive cases those that tested positive to the point-of-care or immunoassay IgG tests [Supplementary Table 6 in Pollán et al. (14), column *Either test positive*].

For our estimates, we consider the (up to) 100 most recent survey responses per region on April 20. The date is chosen because the mean period between illness onset and a 95% confidence of IgG antibodies presence is 14 days (16). This results in *n* = 999 responses (59 ± 35 per region) across Spanish regions, with a cumulative reach of $\underset{i}{\sum }r_{i}=67,199$ (0.1827 ± 0.0701% of the regional population, and 0.1434% of the national population). On average, participants provide information for *r*_*i*_ = 74.6219 ± 38.0291 members in their social circle, which is coherent with Dunbar's acquaintance group and related studies that take social networks into consideration (17). Within this dataset, our outlier removal methods excluded 19.8883 ± 9.2692% of responses, including spurious contributions as the original average reach per participant before filtering was >5 · 10^15^.

**Figure 1:**
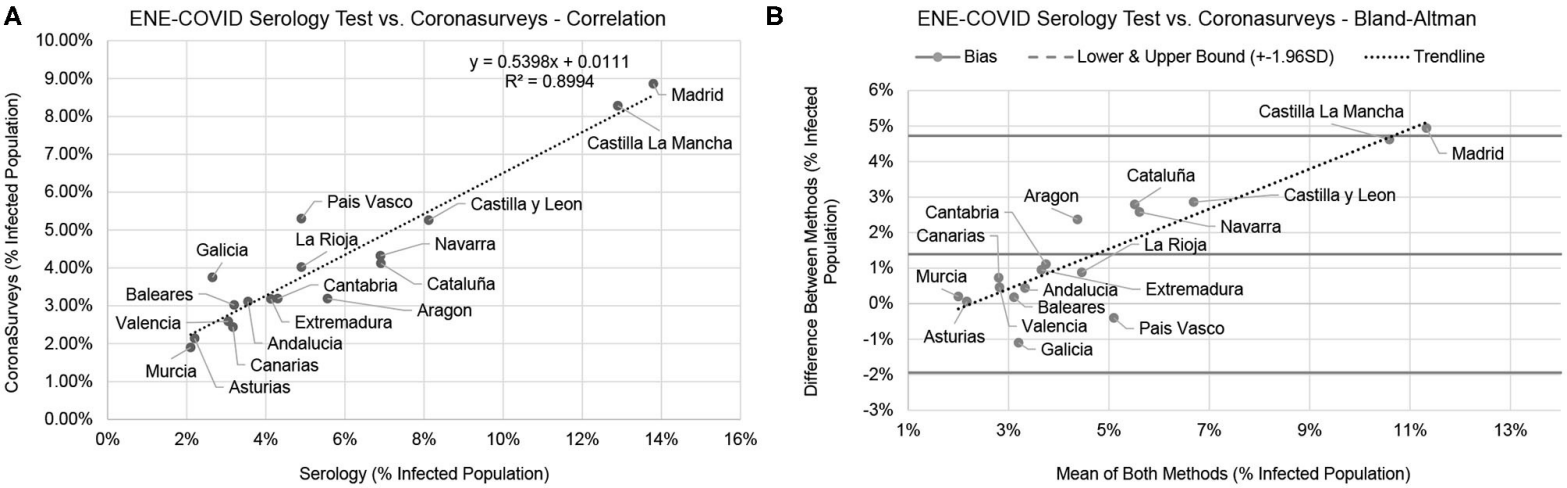
Comparison between the serology test and CoronaSurveys, direct correlation **(A)**, and Bland-Altman **(B)**.

The Bland-Altman plot in Figure 1B shows a high correlation between the CoronaSurveys estimates and the gold standard. A direct comparison of crude percentages, depicted in Figure 1B, also yields excellent results (*R*^2^ = 0.8994). Table 1 presents a detailed comparison of the estimates per region obtained in the different studies.

**Table 1:** Percentage (and 95% confidence interval) of infected population per region according to the ENE-COVID serology study (14), CoronaSurveys, and Covid19Impact (11) (symptom-only model).

	**ENE-COVID**	**CoronaSurveys**			**Covid19Impact**	
**Region**	**% Infected**	**% Infected**	**Responses**	**Reach**	**% Infected**	**Responses**
Andalucia	3.55	3.11 (±0.41)	100	6, 721	2.2 (±0.3)	5, 691
Aragon	5.56	3.19 (±0.41)	44	3, 045	2.0 (±0.3)	1, 463
Asturias	2.20	2.14 (±0.52)	42	2, 987	1.5 (±0.3)	655
Cantabria	4.30	3.19 (±0.96)	16	1, 285	2.8 (±0.3)	497
Castilla y Leon	8.12	5.26 (±0.58)	86	5, 763	3.7 (±0.4)	1, 994
Castilla La Mancha	12.90	8.28 (±0.68)	100	6, 399	8.0 (±0.3)	3, 469
Canarias	3.17	2.44 (±0.74)	26	1, 678	1.4 (±0.2)	1, 052
Catalonia	6.91	4.12 (±0.49)	100	6, 310	2.8 (±0.3)	5, 088
Extremadura	4.13	3.18 (±0.74)	32	2, 168	2.3 (±0.4)	656
Galicia	2.65	3.75 (±0.49)	85	5, 781	1.3 (±0.3)	2, 257
Baleares	3.20	3.02 (±0.76)	33	1, 955	1.9 (±0.3)	1, 222
Murcia	2.10	1.90 (±0.50)	45	2, 835	1.5 (±0.3)	3, 566
Madrid	13.8	8.86 (±0.67)	100	6, 850	6.1 (±0.4)	10, 365
Navarra	6.90	4.32 (±1.16)	16	1, 180	3.6 (±0.4)	580
Basque Country	4.90	5.30 (±0.65)	65	4, 511	1.9 (±0.4)	1, 007
La Rioja	4.90	4.02 (±1.72)	9	498	1.8 (±0.4)	220
Valencia	3.05	2.59 (±0.37)	100	7, 233	1.6 (±0.3)	102, 021

**Figure 2:**
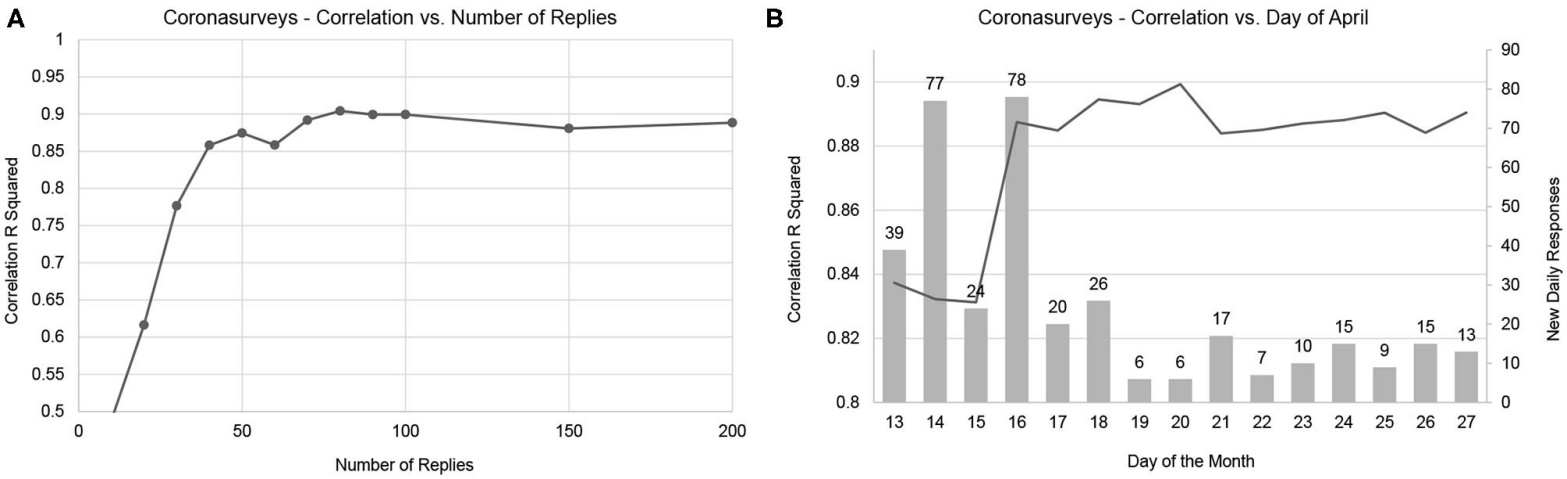
Convergence of correlation with number of responses **(A)** and day of the month **(B)**. The line represents the resulting R squared correlation, the dots in the line represent sampling points. The bars represent the number of new daily responses.

Figure 2A presents how the number of responses per region affects the resulting value of *R*^2^. This analysis indicates that 50 responses per region can already offer a reasonable estimation of cases. Including more responses may further increase accuracy, but the numbers remain reasonably stable. Naturally, it is important that responses are well-distributed across all regions. Figure 2B depicts the effect of the day limit on *R*^2^ if we consider a date of ±1 week. Theoretically, a bell curve centered on the 20th should be expected, as estimating too early would imply too few cases are reported, and estimating too late would include more cases. We indeed observe an impact on accuracy, and the left half of the bell curve is more visible. The change in accuracy is mostly due to new daily responses collected on April 16th. The lack of the right half of the bell curve is due to the low number of new daily responses after April 16th, which implies that the daily estimates are computed with sets of responses with large intersections. Interestingly, a similarly high number of responses was collected on April 14th, with nearly no impact on accuracy.

## 4. Discussion

The linear regression equation in Figure 1A points to CoronaSurveys very consistently underestimating the number of cases by a factor of ~46%, possibly due to asymptomatic cases. This ratio is consistent with the estimates of the Covid19Impact study of Oliver et al. (11), which used more than 140, 000 direct survey responses collected on March 28th–30th. It is also consistent with the reported data on asymptomatic cases reported by Pollán et al. (14), which found that around a third of the seropositive participants were asymptomatic (see Table 1).

**Figure 3:**
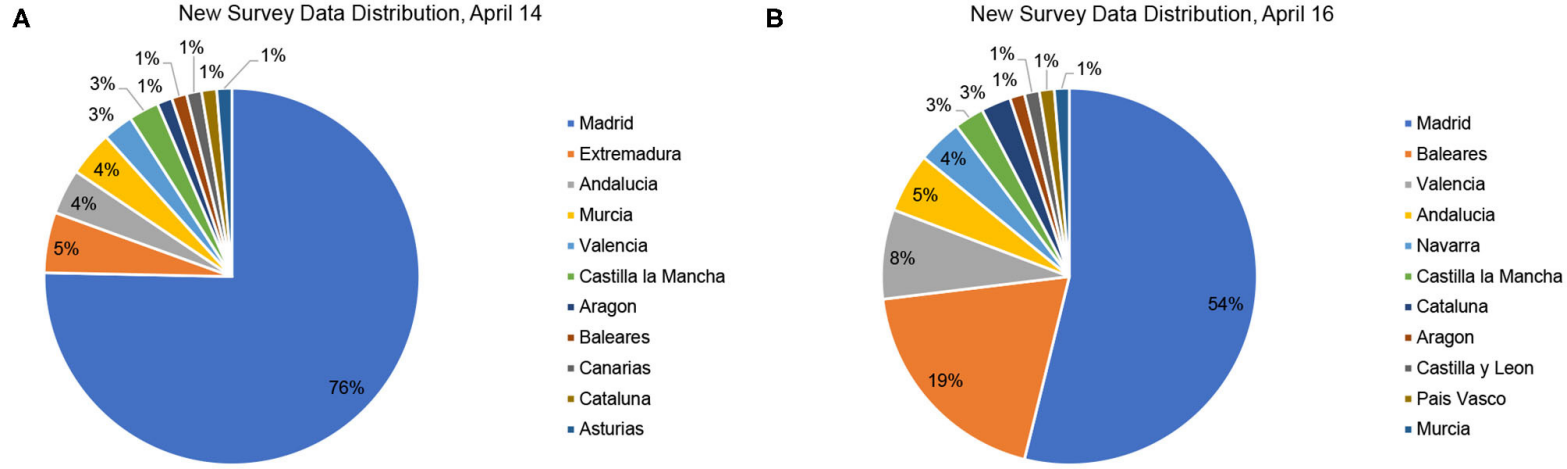
Distribution of new survey responses on April 14 **(A)** and April 16 **(B)**.

Concerning the impact of the number of responses as depicted in Figure 2, we observe how once the minimum number is reached, further responses will not significantly increase accuracy unless these come from underreported regions. As depicted in Figure 3, additional responses from regions where many are already available will barely have an impact on the global result. As the great majority of contributions for April 14th were for Madrid, where we already had many responses available, the 77 new daily responses on April 14th barely had any impact, while the contributions on April 16th significantly increase the accuracy of our estimation.

Our study presents a number of limitations. Firstly, as presented in Table 1, our number of responses in some regions was limited (e.g., nine responses in La Rioja or 16 in Navarra and Cantabria). Our own analysis suggests this is not enough to offer reliable data for these three regions. Additionally, our criteria to eliminate outliers is heuristic, and may change in the future as we collect more data.

Nevertheless, despite these limitations, the estimates obtained in CoronaSurveys show high correlation with serology tests. Moreover, since the underestimation of our method over all regions is homogeneous, and consistent with the one third fraction of asymptomatic reported by Pollán et al. (14), these estimates can be “corrected” to provide an accurate cumulative number of cases for each region. We will further evaluate the robustness of our model as Pollán et al. publish the results of their three additional serology studies.

In summary, we believe these results strongly support using open surveys with indirect reporting as a method to broadly sense the progress of a pandemic.

## Data Availability Statement

The datasets presented in this study can be found in online repositories. The names of the repository/repositories and accession number(s) can be found at: https://github.com/GCGImdea/coronasurveys.

## Ethics Statement

The studies involving human participants were reviewed and approved by IMDEA Networks Ethics Committee. The patients/participants provided their written informed consent to participate in this study.

## Author Contributions

The analysis presented in this article was conducted by AG-A and AF with support and feedback from all remaining co-authors. The data acquisition and processing techniques were developed by all authors.

## Conflict of Interest

NN and ES were employed by the company Algolysis Ltd. The remaining authors declare that the research was conducted in the absence of any commercial or financial relationships that could be construed as a potential conflict of interest.
